# Occupational stress experienced by residents and faculty physicians on night shifts

**DOI:** 10.1186/s13049-016-0225-4

**Published:** 2016-03-22

**Authors:** Feriyde Çalişkan Tür, İbrahim Toker, Cafer Tayyar Şaşmaz, Serkan Hacar, Burcu Türe

**Affiliations:** Department of Emergency Medicine, Izmir Tepecik Training and Research Hospital, Izmir, Turkey; Department of Public Health, Mersin University Faculty of Medicine, Mersin, Turkey; First and Emergency Aid Program and Occupational Health Unit, School of Healthcare Studies, Dokuz Eylül University, Izmir, Turkey

**Keywords:** Occupational health, Physician, Physician’s specialty, Night shift work, Job stress

## Abstract

**Background:**

Occupational stress is an undesired factor causing discomfort for healthcare workers. Stressors in work can lead to dissatisfaction and in turn, this may affect patient care adversely. The aim of this study was to evaluate the occupational stress among residents and faculty physicians of various medical specialties working night shifts.

**Methods:**

Residents and faculty physicians working night shifts in the emergency departments, medical and surgical wards were questioned with Swedish Demand Control Support Questionnaire. Also, various factors (specialty, marital status, sex, number of patients during a typical shift, number of night shifts per month, decision about career making in that specialty, having chronic disease and/or sleep problem) originated from social life or working conditions were investigated that could affect the demand, job-control and job strain model.

**Results:**

Of the 108 participants, the mean age was 31 ± 6 years, 40.7 % were female, and 78.7 % were residents. Job strain was similar among the three physician groups (*p* > 0.05). Job control and social support was found to be lower among residents while job stress was higher. The social support-scores were lower in residents who were responsible for more than 60 patients, and who had a chronic disease. The demand-scores were lower in faculty physicians who worked 1 to 4 night shifts per month. Job strain was higher in residents with respect to faculty physicians.

**Discussion:**

Stress and psychosocial risk factors are considered critical issues in the field of occupational health. Workload and job stress are stated as predictors of workers' health, productivity, and motivation. We found a few job stressors by physician working night shifts such as number of taken care of patient, having chronic disease. But, these physicians were significantly residents, due to their high workload sense. Interestingly, job stress was not more by emergency physicians than others.

**Conclusions:**

Job strain was found to be higher among the residents as compared to the faculty physicians. Job strain was not significantly different among the physicians of emergency medicine than the physicians of the other specialties working night shifts.

## Background

Physician wellness is frequently discussed, though it is not sufficiently prioritized. Variations in health care policies, increasing patient admissions, increasing responsibilities, and environmental stressors can affect physician wellness [1]. A physician’s performance is also partially related to health economics, in the sense that his/her income may depend on ordering and prescribing preferences, and decisions to admit or operate a patient. These decisions may be evaluated by a variety of entities and this may also cause stress. The psychosocial aspects of work have been increasingly discussed in the medical literature in recent years [2–5]. Majority of physicians admit that patient care is affected negatively by their stress at work [3–5]. A physician’s workload involves both cognitive and emotional burdens leading to an increase in job stress (JS) [1, 4, 6, 7]. Implementations with a view to reduce the physicians’ JS should lead to an increase in physicians’ performance and consequently serve for a more satisfactory patient care [1–3, 7–9].

Stress and psychosocial risk factors are considered critical issues in the field of occupational health [1, 2, 10]. Workload and JS are stated as predictors of workers’ health, productivity, and motivation [10]. The most frequently used models to measure JS are those examining the worker’s psychosocial workload (demand) and sense of job control[11–16]. Demand is higher in those working night shifts, especially among emergency physicians [4, 7, 17]. Abnormal stress levels are reached earlier in emergency physicians compared to those of other medical specialties [7, 17]. JS is higher in female physicians, medical students, and residents compared to the general population [16, 17]. Stress is higher in physicians who are dissatisfied with their job, causing 30-50 % of such physicians to change or leave their occupation [4, 7, 17]. The level of JS and sense of job control may differ from one healthcare system to another, and also display variations in different populations. However, social support and time may change the status of the job satisfaction, too. These reasons led us to perform this study where physicians are seperated as residents and faculty physicians from different specialties working night shifts in order to evaluate the problem from workload management perspective by scores of demand, job control, social support, and JS.

## Methods

### Study setting and population

Our cross-sectional study protocol was approved by the Ethics Committee and the research was done in a tertiary care training and research hospital. Questionnaires (see details below) were administered by researchers to participants in their own departments/wards during a rest break while they were working night shifts, in the period between January and February 2014. Each questionnaire took approximately 10 min to complete.

### Selection of the participants

The total number of the physicians working night shift in the study center was 146. Three groups were constituted; emergency medicine, internal medicine and surgical branches. All the emergency residents and emergency faculty physicians on the same duty were enrolled. The physicians of medical and surgical branches were randomized so that the number of physicians of other specialties on duty was equal to the number of the emergency physicians. In the emergency department, all physicians on a given shift were approached for participation (*n* = 37). Specialties and numbers of physicians participating from internal medicine branches (total *n* = 39) were: internal medicine (11), neurology (5), pediatrics (10), radiology (6), cardiology (2), and infectious diseases (2). Specialties and numbers of physicians participating from each surgical specialty were (total *n* = 39): neurosurgery (3), general surgery (12), orthopedics (10), urology (2), ophthalmology (2), anesthesiology (7), and otolaryngology (2). A total of 115 physicians were planned to be included after randomisation. The name list of the physicians participating in the survey was the hospital’s official ‘doctors on duty list" which was prepared independent of this research study, by keeping both the interviewers and participating physicians blinded to the list (double-blind). Two declined to participate, and the questionnaires of five physicians were incomplete, thus data from 108 physicians were evaluated. Emergency medicine physicians worked a 16-h night shift (4 p.m. to 8 a.m.) whereas those from other specialties worked a 32-h shift (8 a.m. until 4 p.m. the next day). Physicians participated voluntarily; those who consented to participate gave demographic data (age, sex, years employed, chronic diseases, intent to work) and then they were administered the Pittsburgh Sleep Quality Index (PSQI) and the Swedish Demand Control Support Questionnaire (DCSQ) [14, 18–20]. This study focuses on the results of the DCSQ; whereas the PSQI results are presented in a separate research paper [21].

### Measures

The DCSQ measures psychosocial JS by 17 questions, which cover three dimensions of job stress [14, 15, 22]: demand (5 questions), job-control (6 questions), and social support (6 questions). Demand is the employee’s perceived workload (tasks required to be performed in the midst of possible workplace conflicts), control is the sense of autonomy about making work decisions or decision latitude (vs. having tasks programmed by one’s employer), and SS is encouragement and support from colleagues and superiors. Job strain refers to the psychological and physiological reactions of the worker to stressors. Participants with high measured job strain are considered to have high levels of job stress [11, 12, 15]. The questions on the questionnaire have four choices from 1 (always, or definitely agree) to 4 points (never, or definitely don’t agree). Sub-section scores were calculated. Job stress was measured as the ratio of demand to job strain [18–20]. This version of the DCSQ was validated by Demiral et al. [14].

### Outcomes

The primary outcome of the study was the comparison of job stress level (Job-strain, Demand, Job-control and social support scores) among residents and faculty physicians by DCSQ. Secondary outcome was the description of the factors (specialty, marital status, sex, patient number cared for during a typical shift, number of night shifts per month, decision about continuing in specialty, having chronic disease and poor sleep quality) that could affect the Job strain, Demand and Job-Control model, and those originated from social life or working conditions. ‘Job strain’ in DCSQ is called generally as ‘job stress’ in this paper.

Another result of our study is the relationship between sleep quality scores determined by the PSQI and JS scores.

### Analysis

Statistics were implemented by using statistical software. Descriptive statistics were used to summarize the data. The homogeneity and normal distribution of the data were assessed by the Kolmogorov-Smirnov test. Kruskal-Wallis and Mann–Whitney U significance tests were used for the comparison of dependent and independent variables. Correlation between residents’ and faculty physicians’ mean age and their job strain scores were assessed by Kendall’s tau correlation test. *P* < 0.05 was considered statistically significant. The relationship between job-stress and poor sleep quality was assessed by DCSQ and PSQI scores.

## Results

A total of 108 physicians were included in the study, 64 (59.3) of these were male. Regarding specialty fields, 37 (34.3 %) physicians worked in emergency medicine, 35 (32.4 %) were in internal medicine and 36 (33.3 %) in surgical branches. The mean age of the participants was 31 ± 6 (range 25–52) years, and 78.9 % were residents. At least one chronic disease were reported by 37 %. Demographic characteristics of the physicians working night shifts vs physicians specialties are listed in Table 1.

**Table 1:** Demographic characteristics of 108 physicians working night shifts by physician’s specialty. Results are given as number (*n*) and mean value or percentage

Variables		Emergency medicine		Surgical specialties		Medical specialties		Total	
		*n*	mean or %	*n*	mean or %	*n*	mean or %	*n*	mean or %
Age		37	32.4 ± 4.8	36	31.7 ± 7.1	35	29.6 ± 5.9	108	31.3 ± 5.9
Man		23	62.2	27	75.0	14	40.0	64	59.3
Woman		14	37.8	9	25.0	21	60.0	44	40.7
Married		25	67.6	17	47.2	14	40.0	56	51.9
Unmarried		12	32.4	19	52.8	21	60.0	52	48.1
Patients cared for on a typica night shiftl	1-30	2	5.4	25	69.4	14	40.0	41	38.0
	31-60	8	21.6	8	22.2	7	20.0	23	21.3
	>60	27	73.0	3	8.3	14	40.0	44	40.7
Night shifts per month	1-4	9	24.3	8	22.2	14	40.0	31	28.7
	5-8	3	8.1	16	44.5	20	57.1	39	36.1
	≥9	25	67.6	12	33.3	1	2.9	38	35.2
Academic status	Resident	28	75.7	29	80.6	28	80.0	85	78.7
	Specialist	9	24.3	7	19.4	7	20.0	23	21.3
Intends to continue in specialty	Yes	21	56.8	22	61.1	26	74.3	69	63.9
	No	16	43.2	14	38.9	9	25.7	39	36.1
Chronic disease and/or sleep problem	Yes	17	45.9	16	44.4	7	20.0	40	37.0
	No	20	54.1	20	55.6	28	80.0	68	63.0
Total n %		37	34.3	36	33.3	35	32.4	108	100.0

### Main results

Demand scores were found higher in the residents than in the faculty physicians and it was statically significant (respectively, 17 and 15, *p* < 0.05). Job-control scores were found to be greater in the faculty physicians compared to the residents (respectively, 18 and16, *p* < 0.001). JS was higher in the residents compared to the faculty physicians (respectively 1.1 and 0.9, *p* < 0.001) There was no statistically significant difference in terms of social support scores between the residents and the faculty physicians (Table 2).

**Table 2:** Median scores for demand, job-control, and social support and calculated total scores for job strain by residents and faculty physicians

Variables	Demand		Job-control		Social support		Job strain	
	Median (min-max)	*P*	Median (min-max)	*p*	Median (min-max)	*p*	Median (min-max)	*P*
Residents (*n* = 85)	17 (6–20)	<**.05**	16 (5–20)	<**.001**	17 (6–24)	>.05	1.1 (0.6-1.7)	<**.001**
Faculty Physicians (*n* = 23)	15 (13–19)		18 (10–20)		18 (6–24)		0.9 (0.7-1.4)	
Total	16 (6–20)		16 (5–20)		17 (6–24)		1.0 (0.6-1.7)	

Job strain and demand was higher, job control was lower in residents than in faculty physicians and these differences were statistically significant

Social support were statistically significantly lower among residents who typically take care of more than 60 patients during a night shift (*p* < 0.05) Social support scores were statistically significantly lower among residents who had a chronic disease (*p* < 0.05). The scores of the residents obtained for other factors such as their specialty, marital status, sex, number of night shifts per month, decision about continuing in their specialty were not statistically significant (*p* > 0.05). Statistically significant difference was not found between specialties of the residents and job strain, job control, demand and social support (Table 3).

**Table 3:** Median scores for demand, job-control, and social support and calculated total scores for job strain versus demographic variables for residents (*n* = 85)

Variables		Demand		Job control		Social support		Job strain	
		Median (min-max)	*p*	Median (min-max)	*p*	Median (min-max)	*p*	Median (min-max)	*P*
Specialty^a^	EM	16 (6–20)	>.05	17 (5–19)	>.05	16.5 (6–24)	>.05	0.9 (0.8-1.4)	>.05
	SURG	17 (10–19)		16 (10–20)		17 (8–23)		1.1 (0.6-1.6)	
	MED	17 (10–19)		16 (10–20)		17 (8–23)		1.1 (0.6-1.6)	
Marital status	Married	16 (6–20)	>.05	16 (5–19)	>.05	17 (6–23)	>.05	1.1 (0.6-1.5)	>.05
	Unmarried	17 (10–20)		16 (10–20)		17 (8–24)		1.1 (0.6-1.7)	
Sex	Man	17 (6–20)	>.05	16 (5–20)	>.05	18 (6–24)	>.05	1.1 (0.6-1.7)	>.05
	Woman	16 (10–20)		16 (10–20)		17 (8–23)		1.1 (0.6-1.7)	
Number of patients cared for during a typical shift	1-30	17 (10–20)	>.05	16 (12–20)	>.05	18 (11–24)	**<.05**	1.0 (0.6-1.7)	>.05
	31-60	16.5 (13–20)		15.5 (12–20)		18 (11–24)		1.1 (0.9-1.4)	
	>60	17 (6–20)		16 (5–20)		16 (6–20)		1.1 (0.8-1.6)	
Number of night shifts per month	1-4	17 (10–19)	>.05	16 (12–20)	>.05	16 (8–23)	>.05	1.0 (0.6-1.2)	>.05
	5-8	17 (11–20)		15 (10–20)		17 (12–23)		1.1 (0.7-1.6)	
	>8	16 (6–20)		16 (5–19)		17 (6–24)		1.0 (0.8-1.7)	
Decided about continuing in specialty	Yes	17 (6–20)	>.05	16 (5–20)	>.05	17 (6–24)	>.05	1.1 (0.6-1.7)	>.05
	No	16 (11–20)		15 (10–19)		18 (11–24)		1.1 (0.6-1.7)	
Chronic disease	Yes	16 (6–20)	>.05	16 (5–19)	>.05	16 (6–21)	**<.05**	1.1 (0.8-1.7)	>.05
	No	17 (10–20)		16 (10–20)		17 (8–24)		1.1 (0.6-1.7)	

Social support was lower in residents who cared for more than 60 patients during a night shift and who had a chronic disease, and these differences were statistically significant

^a^Emergency medicine = EM, Internal medicine specialties = MED and Surgical specialties = SURG

Among faculty physicians, who worked 1–4 night shifts per month reported higher work load (demand) than those who had more night shifts and this difference was statistically significant (*p* < 0.05).

The faculty physicians who reported any chronic disease displayed statistically significantly better job control with respect to the other healthy physicians (*p* < 0.05). JS scores of faculty physicians were not statistically different for the other factors such as specialty, marital status, sex, number of patients in a typical shift and decision about continuing in specialty (*p* > 0.05). No statistically significant difference was detected for the scores of demand, job control, social support, and calculated JS among faculty physicians working in emergency departments or medical or surgical wards (*p* > 0.05, Table 4).

**Table 4:** Median scores for demand, job-control, and social support and calculated total scores for job strain versus demographic variables for faculty physicans (*n* = 23)

Variable		Demand		Job-control		Social support		Job strain	
		Median (min-max)	*P*	Median (min-max)	*p*	Median (min-max)	*p*	Median (min-max)	*P*
Specialty^a^	EM	15 (14–18)	>.05	18 (16–20)	>.05	18 (16–22)	>.05	0.8 (0.8-1.1)	>.05
	SURG	15 (13–19)		18 (10–20)		18 (15–20)		0.8 (0.7-1.4)	
	MED	15 (14–18)		18 (14–19)		19 (6–24)		0.9 (0.7-1.1)	
Marital status	Married	15 (13–19)	>.05	18 (10–20)	>.05	18 (6–24)	>.05	0.8 (0.7-1.4)	>.05
	Unmarried	15.5 (14–18)		17 (14–19)		16.5 (16–21)		1.0 (0.7-1.1)	
Sex	Man	15 (13–19)	>.05	18 (10–20)	>.05	18 (6–24)	>.05	0.8 (0.7-1.4)	>.05
	Woman	16 (14–18)		18 (16–20)		17 (16–19)		0.9 (0.7-1.1)	
Number of patients cared for during a typical shift	1-30	15 (13–19)	>.05	18 (10–20)	>.05	18 (15–22)	>.05	0.9 (0.7-1.4)	>.05
	31-60	15 (14–18)		19 (16–20)		18 (6–22)		0.8 (0.7-1.1)	
	>60	15 (14–17)		18 (16–20)		18 (16–24)		0.8 (0.8-1.1)	
Night shifts per month	1-4	15.5 (14–19)	**<.05**	18 (10–20)	>.05	18 (6–24)	>.05	0.9 (0.7-1.4)	>.05
	5-8	14 (13–15)		19 (14–19)		20 (16–21)		0.7 (0.7-1.1)	
	>8	14.5 (14–15)		17.5 (17–18)		18 (17–19)		0.8 (0.8-0.8)	
Decided about continuing in specialty	Yes	16 (15–19)	>.05	18 (16–20)	>.05	17.5 (6–24)	>.05	0.9 (0.8-1.1)	>.05
	No	15 (13–18)		18 (10–20)		18 (15–22)		0.8 (0.7-1.4)	
Chronic disease	Yes	15 (13–19)	>.05	18.5 (17–20)	**<.05**	17.5 (6–22)	>.05	0.8 (0.7-1.0)	>.05
	No	15 (14–18)		16 (10–20)		18 (16–24)		1.1 (0.7-1.4)	

Job strain was statistically significantly higher in faculty physicians worked 1–4 night shifts a month. Job control was statistically significantly higher in faculty physicians who had a chronic disease

^a^Emergency medicine = EM, Internal medical specialties = MED and Surgical specialties = SURG

We found no statistically significant correlation between ages and DCSQ subcomponet scores of the residents. Among faculty physicians, however, increased age showed a weak positive correlation with job control and a weak negative correlation with JS, which were statistically significant (*p* < 0.01, Table 5).

**Table 5:** Correlation between demand, job control, social support and job strain and age of the residents and faculty physicians

	Demand,p	Job-control, p	Social support, p	Job strain, p
Resident age	k = 0.05,>.05	k = 0.001,>.05	k = 0.04,>.05	k = 0.007,>.05
Faculty physician age	k = −0.09,>.05	k = 0.2,**<.01**	k = 0.08, >.05	k = −0.2,**<.01**

Statistically significant correlation was not detected between the age and demand, job control, social support and job strain, for the residents. Statistically significant weak positive correlation was detected between the age and job control, and a weak negative correlation between age and job strain, for the faculty physicians

We found no relationship between demand, job control, social support and job-stress score and poor sleep quality, either by residents and faculty physicians (all of *p* > 0.05).

## Discussion

In most healthcare environments, professionals in the health sector suffer heavy workload. Overall, work was found to be the biggest (74 %) stressor in the lives of employees [22]. Occupational environment and the nature of the work of the physicians is physically demanding. Long-term occupational stress has been shown to lead to burnout and the term ‘burnout’ is often used by the health care personel synonymously with job stress [4–9, 16]. In conclusion, the aim of this study was to assess occupational stress among residents and faculty physicians in different medical specialties working night shifts. We determined that job control and social support scores were lower but job strain scores were higher among the residents compared to the faculty physicians. This is an expected result and it may be related with the working conditions of the residents. In residence, professional knowledge and skills are imperfect and job-control is low, because the required specialization period is not completed yet. In this sense, residents require assistance that will guide them and support them to improve their knowledge and skills. Previous investigations have determined that seniority, working with consultants in emergency medicine, and feeling appreciated in work place are factors in resident’s satisfaction or burnout status [7, 8, 16, 23]. These are the notions that are synonymous with JS and support the results of our research.

Emergency physicians (EP), who already might have suffered from some degree of sleep deprivation, experience stress from both noncritical and critical patients who demand intense focus and rapid decision-making [1, 4, 7, 24, 25]. They may have cognitive and emotional burdens and even more unrelaxed night shifts than other specialists. For these reasons, the perceived workload and JS of emergency physicians are expected to be higher than other physicians. However, in this study, no difference was detected in demand, job control, social support and JS scores between residents and faculty physicians working in emergency, internal medicine, and surgical branches. JS was not found to be greater in emergency physicians, perhaps due to their increased job-control (they did not reach statistical significance though). Another factor may have been the shorter shift length of the EPs (16 vs. 32 h). However, an EP is far more likely to take care of more than 60 patients in a night shift with minimal relaxing time. This is totally half the length than their colleague’s. Therefore, we consider that JS experienced by EPs may be at least the same as the physicians of the other specialties. Overall, in an environment with high psychological and physiological demands, night shift work can be stressful for the physicians in all specialties and JS displayed no differences between specialities [15, 22]. Our results too, showed that physicians of different specialties who work night shifts frequently face stress factors like JS. It is stated in the literature that if a simple defect in a health unit is not healed timely then it may have an impact on the other units and eventually turn into a general fatigue and dissatisfaction [4, 26–28]. Hence it should be considered that there might be a similar and general JS in all the physicians working night shifts.

Demands reflects work speed, work intensity, and workload [11, 15]. Interestingly, we determined that the demand score of faculty physicians was affected only by the small number of night shifts per month. Demand scores may be so high because of the difficulty of adaptation in case night shifts are rare. Thus, the physicians who are not used to night works may perceive working hours relatively prolonged and this may adversely influence their decision making abilities, hence increase their JS. Faculty physicians included in this study might have perceived higher demand despite they were favored and given very few night shifts, possibly because of a chronic disease or sleep problem or advanced age. So, these faculty physicians may be in the position of increasing their job controls. Nonetheless, we can state that, this demand in the faculty physicians was less with respect to the residents, in our study. As for the residents, however, we found no factors influencing demand; which suggests that it should be a consequence of compulsory submission which is a normally expected of residents.

Job-control reflects the employee’s opportunities to make decisions and how to use his/her skills in the workplace. In jobs with heavy demand and low job-control, stress levels are very high [10, 12, 18]. In situations where JC is high, workers are more creative, more motivated and feel less stressed when faced with heavy workloads [10, 12, 18]. Passive work situations are those in which both demand and job-control are low. In such cases, the time needed for learning skills is reduced, and with low job-control, learned helplessness may occur [10, 12]. Job-control is higher in a work that requires frequent repetition [10, 12, 13, 29, 30]. Occupations characterized by low demand and high job-control are considered low-stress jobs [12, 31]. In the studies using demand, job-control and social support models, white-collar workers were found to have much more job-control than blue-collar workers [12, 31, 32]. In this study, job-control scores were significantly higher in faculty physicians who had a chronic disease. This may be thought as a compensatory mechanism, where a specialist physician feeling inadequate may act highly controlled among residents and their colleagues with a view to become influential or to come into prominence. A positive correlation between age and job-control is an expected result among faculty physicians, due to confidence as a result of professional experience. In residents, however, job-control is normally low due to lack of professional knowledge and skills.

The social environment in a workplace is an independent variable for occupational stress – the worst situations are those where social support is lacking, workload is high, and job-control is low [12, 14, 20, 21]. Many studies have shown that more and more physicians wish to change their specialty and don’t intend to continue their occupation [3, 4, 7, 14, 32–34]. In this study, we couldn’t determine any factor influencing social support score of the faculty physicians, whereas we determined widespread lack of social support among residents. The social support score of the residents who typically see too many patients were found to decrease. Because satisfaction from work and work environment may decrease as a result of automatisation at work and having probably no concerns other than completing the work timely and consequently, this may alienate residents from their profession. This result highlights the urgent need to improve conditions in the work environment of the residents. Nonetheless, we found no relation between the decision to continue in the profession and social support for the physicians, in general.

Some studies reported a relationship between JS and chronic diseases (e.g. sleep disorders, myocardial infarction, cancer, burnout). [14, 20, 22, 28, 33–35] When physician wellness is compromised, increased job stress, burnout, depression, relationship issues, substance abuse, and suicide may occur [1, 4]. Interleukin-8 was measured as a stress biomarker in emergency physicians after working a 24-h shift, and increased levels were found, indicating an increase in inflammatory processes [28]. Emergency physicians working in shifts, along with older physician age, were found to have an increased risk of cardiovascular diseases [35].

Contrary to these findings, we found no relationship between the chronic diseases and the calculated level of JS of residents as well as faculty physicians. Nonetheless, as it is discussed above, having a chronic disease in residents may independently affect JS in advanced stages, due to the reduced social support or is it because chronic diseases (including sleep disorder) occur as a result of having limited social support; this issue deserves/requires further discussion.

A previous study demonstrated that 84.3 % of the night shift physicians did not state any complaints of sleeping problem but 83.3 % of them have poor sleep quality [21]. Still, this study found no relationship between job-stress and poor sleep quality. Perhaps physicians deny their health problems and/or have become completely accustomed to the stressful work environment in which they find themselves. Relationship between sleep problem and job stress needs further investigation.

### Limitations

Because this study was conducted in a single tertiary care teaching hospital, our results may not be generalized. Since there is no cut-off value defined in DCSQ measurements, each institution must make its own assessment of the repeated measurements. Reassessment of the duty physician’s job-stress with DSCQ is recommended after a while for measuring the improvements of subsequent developments after wellness activities, especially by social supports in work place. Administering the questionnaire to those working day shifts may also reveal different results, and might separate out other stress factors from the stress of night shift work.

## Conclusions

As a result, job stress is higher in residents than in faculty physicians. We suggest that, the problems of the work environments of the residents should be identified and concrete actions should be taken in order to improve their working conditions and include them in the decision making mechanisms. Also, it is advisable to pay attention to social support of the residents in the work environment who have a chronic disease or too many patients routinely, in order to reduce their JS.

JS level was similar among emergency physicians and physicians in different specialities working night shifts. In fact, similarity suggest that JS is not high indeed. Reassessment should be done to determine the effects of JS on physician wellness, longevity of career, and patient care outcomes.

## Appendics

### DCSQ in our language version

#### İş yükü-kontrol-destek ölçeği

- İş yükü
  - 1. İşinizde çok hızlı çalışmak zorunda mısınız? (hızlı çalışma)
  - 2. İşinizde çok yoğun çalışmak zorunda mısınız? (yoğun iş)
  - 3. İşiniz çok fazla kuvvet (efor) gerektirir mi? (çaba)
  - 4. İşinizde, işinizle ilgili görevleri yetiştirecek kadar zamanınızoluyor mu? (zaman)
  - 5. İşinizde sizden birbiriyle çelişen görevler istenir mi? (çelişkili iş)
- İş kontrolü
  - Beceri kullanımı
    - 6. İşinizde yeni şeyleri öğrenme olasılığı var mıdır? (öğrenme)
    - 7. İşiniz yüksek düzeyde beceri veya uzmanlık gerektirir mi?(uzmanlık)
    - 8. İşinizde sizden yenilikler yapmanız beklenir mi? (yaratıcılık)
    - 9. İşinizde her gün aynı şeyleri mi yaparsınız? (tekrarlayan iş)
  - Karar serbestliği
    - 10. İşinizi NASIL yapacağınız konusunda karar vermede sizin seçim hakkınız var mı? (işin nasıl yapıldığı)
    - 11. İşinizde NE yapacağınıza karar vermede sizin seçim hakkınız var mıdır? (ne yapıldığı)
- Sosyal destek
  - 12. Çalıştığım yerde sakin ve hoş bir ortam var (hoş ortam)
  - 13. Çalıştığım yerde birbirimizle iyi geçiniriz (iyi geçinme)
  - 14. İş yerinde diğer çalışanlar beni destekler (çalışma arkadaşlarının desteği)
  - 15. Kötü günümdeysem iş yerindekiler durumumu anlarlar (çalışma arkadaşlarından anlayış)
  - 16. Üstlerimle ilişkilerim iyidir (süpervizör desteği)
  - 17. İş arkadaşlarımla çalışmak hoşuma gider (çalışma arkadaşlarından hoşnut)
